# Patients with Terminal Interstitial Pneumonia Require Comparable or More Palliative Pharmacotherapy for Refractory Dyspnea than Patients with Terminal Lung Cancer

**DOI:** 10.1089/pmr.2021.0010

**Published:** 2021-06-16

**Authors:** Hiroko Okabayashi, Hideya Kitamura, Satoshi Ikeda, Akimasa Sekine, Tsuneyuki Oda, Tomohisa Baba, Eri Hagiwara, Takuro Sakagami, Takashi Ogura

**Affiliations:** ^1^ Department of Respiratory Medicine, Kanagawa Cardiovascular and Respiratory Center, Yokohama City, Japan.; ^2^ Department of Respiratory Medicine, Kumamoto University Hospital, Faculty of Life Sciences, Kumamoto University, Kumamoto City, Japan.

**Keywords:** interstitial pneumonia, lung cancer, midazolam, morphine, palliative care, palliative sedatives

## Abstract

***Background:*** Dyspnea is a severe symptom of terminal-stage interstitial pneumonia (IP). We commonly use continuous morphine or midazolam for terminal refractory dyspnea.

***Objective:*** We aimed to determine whether there is a difference in the use of continuous morphine and midazolam for terminal dyspnea between IP patients and lung cancer (LC) patients.

***Design:*** This is a single-center retrospective study.

***Setting/Subjects/Measurements:*** We retrospectively examined the clinical records of IP and LC patients who had died in our hospital. These patients were divided into the IP and LC groups to compare the use of morphine and midazolam.

***Results:*** Continuous morphine was administered to 50.0% of those in the IP group and 38.0% of those in the LC group for terminal dyspnea. There was no difference in the effect at six hours after morphine initiation between the two groups, but the concomitant use of continuous midazolam and morphine was more common in the IP group than in the LC group. The dose of continuous midazolam was significantly higher in the IP group than in the LC group, and the survival time after morphine initiation was significantly shorter in the IP group.

***Conclusions:*** The efficacy of continuous morphine administration for terminal dyspnea in IP patients was similar to that in LC patients for a short time after initiation, but just before death, more patients in the IP group required concomitant use of midazolam and morphine. Thus, IP patients require comparable or more palliative treatment than LC patients.

## Introduction

Patients with certain types of fibrosing interstitial lung diseases (ILDs), such as idiopathic pulmonary fibrosis (IPF), are at risk of respiratory symptoms aggravation, lung function decline, limited therapeutic response, decreased quality of life, and early mortality.^1–3^ Dyspnea and cough are very common symptoms in interstitial pneumonia (IP) patients. In a nationwide registry-based cohort study of patients with oxygen-dependent ILD and patients with lung cancer (LC) in Sweden, ILD patients had a significantly longer period of breathlessness and lower rates of complete relief from breathlessness than LC patients.^4^

Morphine is reportedly effective for refractory dyspnea in patients with cancer, with the guidelines recommending its use.^5,6^ Several statements and guidelines have addressed the use of morphine for end-stage dyspnea in nonmalignant respiratory diseases.^7–9^ Palliative sedation is a therapeutic option in cases wherein symptom relief is difficult to achieve.^10^ Midazolam has been mentioned as the first-choice drug for palliative sedation in several guidelines.^11–14^

To date, no studies have described differences in palliative pharmacotherapy for refractory dyspnea in terminal stages of IP and LC. In this study, we determined whether there is a difference between the use of continuous morphine and midazolam for relieving terminal dyspnea in IP patients and LC patients.

## Methods

### Patients

We retrospectively reviewed the medical records of IP and LC patients who had died at Kanagawa Cardiovascular and Respiratory Center from January 2015 to December 2017. The exclusion criteria were as follows: (1) treatment with endotracheal intubation and ventilation, (2) active malignancy other than LC, and (3) concomitant IP and LC.

Patients who received continuous morphine for terminal dyspnea relief were divided into the IP group and LC group. The patients who received continuous opioids for pain control were excluded.

### Continuous morphine and midazolam administration

At our institution, continuous morphine is used for patients with terminal dyspnea that is difficult to relieve through treatment of underlying diseases or oral opioids. If dyspnea is severe and requires continuous deep sedation, continuous midazolam is used. Continuous deep sedation is defined as the continuous use of sedative medication to relieve intolerable and refractory distress by achieving almost or complete unconsciousness until death. The timing of initiation for these drugs was at the discretion of each physician. These drugs were administered subcutaneously or intravenously. Oral opioids were used to relieve dyspnea or pain before continuous morphine was administered.

### Outcomes

We consulted the patients' medical records and collected information regarding their characteristics, periods of treatment from the initial continuous morphine administration to death, the initial and maximum doses of morphine, the use of continuous midazolam, and the use of oral opioids before initiating continuous morphine.

We evaluated the efficacy of continuous morphine at 6 hours after morphine initiation in patients who survived for >12 hours after morphine initiation because it is difficult to evaluate the effect of the drug just before death. This was a retrospective study, and we did not use uniform subjective and objective scores for assessing dyspnea. Thus, based on the medical records, if any of the following items were met, it was judged to be effective: (1) the patients said “dyspnea is relieved” or “I feel better than before,” (2) the family said that the patient's dyspnea seemed to be resolving, (3) physician or nursing records described that the patients had no distress-like expression, and (4) the use of rescue to relieve dyspnea was not necessary. We considered it not effective if none of them were met.

Consent for the use of continuous morphine and midazolam was obtained from the patients or their families. We obtained ethical approval for our study from the institutional review board of the Kanagawa Cardiovascular and Respiratory Center (KCRC-20-0048).

### Statistical analyses

Continuous variables are expressed as median values. The Mann–Whitney *U* test, chi-square test, or Fisher's exact probability test was used for comparing the variables between the two groups. A *p*-value <0.05 was considered to indicate statistical significance. All statistical analyses were performed using the Statistical Package for the Social Sciences version 24.0 (IBM, Armonk, NY).

## Results

**FIG. 1.**
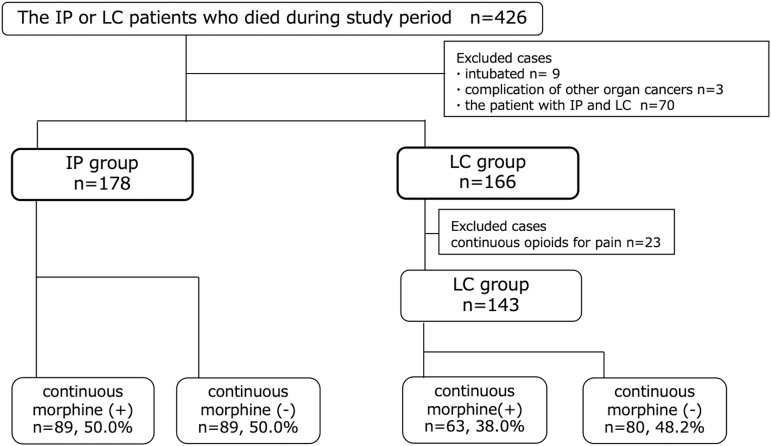
Patient flow diagram. IP, interstitial pneumonia; LC, lung cancer.

Four hundred twenty-six patients with IP or LC died during the study period. Of these, 12 were excluded because of intubation or complications from other active organ cancers, and 70 patients with both IP and LC were excluded. Twenty-three patients in the LC group were treated with continuous opioids to achieve pain relief (Fig. 1). Continuous morphine was administered to 50.0% (89/178) of those in the IP group and 38.0% (63/166) of those in the LC group to relieve dyspnea at the end of life. The IP group was significantly more likely to require medication for relief of terminal dyspnea (*p* = 0.02).

Table 1 gives the characteristics of the patients who were treated with continuous morphine for relief of terminal dyspnea. IPF was the most common IP (52/89 cases) in the IP group. At the initiation of continuous morphine administration, 71.9% of patients had acute exacerbation of IP. The IP group was treated with long-term oxygen therapy (LTOT) before the last hospitalization more often than the LC group. SpO_2_/FiO_2_ at continuous morphine initiation was significantly lower in the IP group than in the LC group. The median initial dose of continuous morphine was 9.6 mg per day in both groups. The median maximum doses of continuous morphine in the LC group were higher than those in the IP group. Concomitant use of continuous midazolam and morphine tended to be more common in the IP group, and the maximum doses of continuous midazolam were significantly higher in the IP group than in the LC group (*p* = 0.03).

**Table 1.** Characteristics of Patients Who Received Continuous Morphine Administration

	IP group *n* = 89	LC group *n* = 63	*p*
Age, years	75 (43–90)	71 (47–97)	0.10
Male gender	68 (76.4%)	40 (63.5%)	0.08
Follow-up period from the first visit, days	1132 (5–6711)	503 (11–3128)	0.00
Smoking history, past/never	59/29	49/14	0.15
BMI	21.6 (12.8–29.8)	19.4 (13.1–31.6)	0.00
Diagnosis of IP			
IPF	52 (58.4%)	—	
Non-IPF IIPs	26 (29.2%)	—	
Connective tissue disease-related IP	9 (10.1%)	—	
Fibrotic hypersensitivity pneumonitis	2 (2.2%)	—	
Acute exacerbation of IP^a^	64 (71.9%)	—	
Use of oral opioids^b^	19 (21.3%)	37 (58.7%)	0.00
LTOT^c^	69 (77.5%)	16 (25.4%)	0.00
HFNC oxygen^a^	22 (24.7%)	1 (1.6%)	0.00
NPPV^a^	14 (15.7%)	0	0.00
SpO_2_/FiO_2_^a^	93.0 (41.0–383.3)	155.0 (60.0–471.4)	0.00
Continuous morphine			
Initial dose, mg/day	9.6 (1.2–48.0)	9.6 (2.4–28.8)	0.05
Maximum dose, mg/day	14.4 (1.2–192.0)	19.2 (8.4–192)	0.03
Administration period, days	2 (0–22)	3 (0–32)	0.01
Continuous midazolam	12 (13.5%)	3 (4.8%)	0.07
Initial dose, mg/day	19.2 (9.1–48.0)	12.0 (9.6–18.0)	0.19
Maximum dose, mg/day	28.8 (9.6–76.8)	18.0 (9.6–24.0)	0.03

The data are presented as the median (range) or frequency (percentage) values.

^a^ At the initiation of continuous opioid administration.

^b^ Use of opioids before continuous morphine administration.

^c^ Before the last hospitalization.

BMI, body mass index; HFNC, high-flow nasal cannula; IIPs, idiopathic interstitial pneumonias; IP, interstitial pneumonia; IPF, idiopathic pulmonary fibrosis; LC, lung cancer; LTOT, long-term oxygen therapy; NPPV, noninvasive positive pressure ventilation; SpO_2_/FiO_2_, oxygen saturation of peripheral artery/fraction of inspiratory oxygen.

The median survival durations after the initiation of continuous morphine were two days in the IP group and three days in the LC group. The IP group had significantly shorter survival duration after the initiation of continuous morphine than the LC group.

**FIG. 2.**
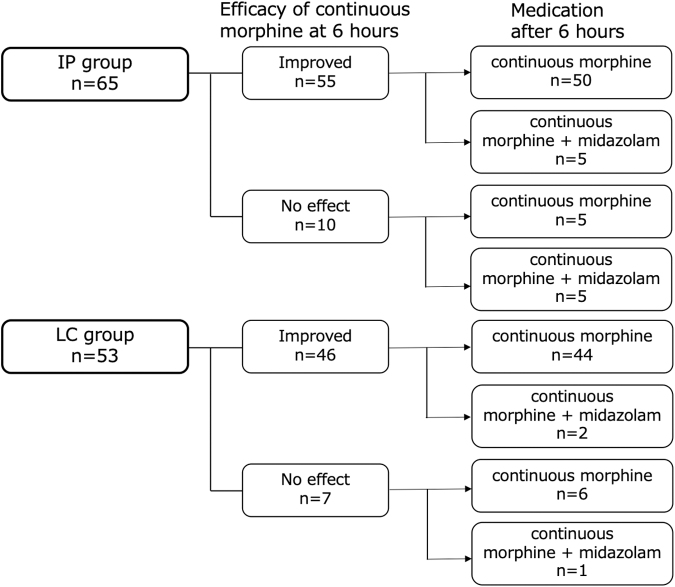
Efficacy of continuous morphine at six hours after morphine initiation and medication after six hours in patients who were surviving at >12 hours after morphine initiation.

The number of patients who were surviving at >12 hours after morphine initiation was 65 in the IP group and 53 in the LC group (Fig. 2).

After morphine initiation, 84.6% (55/65) of those in the IP group and 86.8% (46/53) of those in the LC group showed reduction in terminal dyspnea, representing a statistically insignificant difference. Dyspnea was relieved by six hours after the initiation of continuous morphine, but dyspnea became more severe after six hours, and continuous midazolam was required in five patients (5/55, 9.1%) in the IP group and two patients (2/46, 4.3%) in the LC group.

## Discussion

This study is the first report comparing palliative medication for end-stage dyspnea in IP and LC. We found that the effect of continuous morphine administration for relief from terminal dyspnea was similar in IP and LC patients at six hours after morphine initiation, but more patients in the IP group required additional continuous midazolam because continuous morphine alone did not relieve dyspnea just before death.

In this study, the IP group had a higher rate of LTOT use and lower SpO_2_/FiO_2_ at the start of continuous morphine administration than the LC group. The IP group had worse respiratory function just before death than the LC group, and their dyspnea may have been more severe, requiring more medication for relief from terminal dyspnea.

Opioids are the primary pharmacologic treatment for refractory dyspnea in patients with respiratory diseases.^9^ The efficacy of sustained-release morphine or diamorphine in patients with refractory dyspnea and predicted prognosis of one month or more were reported in the study on respiratory diseases including ILD.^15,16^ In a study that evaluated the safety of sustained-release opioids in ILD with LTOT, opioids were not associated with increased hospital admissions or mortality.^17^ Three reports describe the efficacy and safety of continuous morphine administration in terminally ill IP patients.^18–20^

This study revealed that in some patients, especially in those with IP, it was challenging to achieve dyspnea relief with morphine monotherapy at the end of life. In such cases, continuous evaluation of the need for palliative sedation, rather than increases in the morphine dose, is recommended if death is unavoidable. A single-center study demonstrated that the addition of midazolam to morphine reduced dyspnea in patients with advanced malignancies and an expected prognosis of ≤1 week.^21^

This study has certain limitations. First, this was a retrospective study, and this may include some biases. The initiation of continuous morphine or midazolam was at the discretion of each physician. Second, we did not use uniform subjective and objective scores for patient evaluation. We performed a retrospective evaluation of the medical records to determine whether there was any reduction in dyspnea with continuous morphine, but we were unable to assess the degree of improvement.

## Conclusion

The efficacy of continuous morphine for end-stage dyspnea was similar in IP and LC patients for a short time after treatment initiation. However, just before death, more IP patients required continuous midazolam. Our findings demonstrated that IP patients require comparable or more palliative treatment than LC patients.

## Ethics Approval

The institutional review board of Kanagawa Cardiovascular and Respiratory Center in Kanagawa, Japan, approved the study protocol.

## Availability of Data and Material

The dataset supporting the conclusions of this article is presented in the article. The detailed clinical data are not available due to patient confidentiality.

## Abbreviations Used

BMI: body mass index
HFNC: high-flow nasal cannula
IIPs: idiopathic interstitial pneumonias
ILD: interstitial lung disease
IP: interstitial pneumonia
IPF: idiopathic pulmonary fibrosis
LC: lung cancer
LTOT: long-term oxygen therapy
NPPV: noninvasive positive pressure ventilation
SpO_2_/FiO_2_: oxygen saturation of peripheral artery/fraction of inspiratory oxygen
